# Greater Breast Support Alters Trunk and Knee Joint Biomechanics Commonly Associated With Anterior Cruciate Ligament Injury

**DOI:** 10.3389/fspor.2022.861553

**Published:** 2022-05-20

**Authors:** Hailey B. Fong, Alexis K. Nelson, Julie E. Storey, Jay Hinton, Melissa Puppa, Deirdre McGhee, Daniel Greenwood, Douglas W. Powell

**Affiliations:** ^1^Breast Biomechanics Research Center, College of Health Sciences, University of Memphis, Memphis, TN, United States; ^2^Department of Orthopaedics and Biomedical Engineering, College of Medicine, University of Tennessee Health Science Center, Memphis, TN, United States; ^3^Breast Research Australia, University of Wollongong, Wollongong, NSW, Australia

**Keywords:** ACL, breast, knee, sports bra, biomechanics, landing, injury

## Abstract

**Objective:**

The female breast is a passive tissue with little intrinsic support. Therefore, women rely on external breast support (sports bras) to control breast motion during athletic tasks. Research has demonstrated that lower levels of breast support are associated with altered trunk and pelvis movement patterns during running, a common athletic task. However, no previous study has identified the effect of sports bra support on movement patterns during other athletic tasks including landing. Therefore, the purpose of this study was to examine the effects of breast support on trunk and knee joint biomechanics in female collegiate athletes during a double-leg landing task.

**Methods:**

Fourteen female collegiate athletes completed five double-leg landing trials in each of three different sports bra conditions: no support, low support, and high support. A 10-camera motion capture system (250 Hz, Qualisys, Goteburg, Sweden) and two force platforms (1,250 Hz, AMTI, Watertown, MA, USA) were used to collect three-dimensional kinematics and ground reaction forces simultaneously. Visual 3D was used to calculate trunk segment and knee joint angles and moments. Custom software (MATLAB 2021a) was used to determine discrete values of dependent variables including vertical breast displacement, knee joint and trunk segment angles at initial contact and 100 ms post-initial contact, and peak knee joint moments. A repeated measures analysis of covariance with *post-hoc* paired samples *t*-tests were used to evaluate the effect of breast support on landing biomechanics.

**Results:**

Increasing levels of breast support were associated with reductions in peak knee flexion (Right: *p* = 0.008; Left: *p* = 0.029) and peak knee valgus angles (Right: *p* = 0.011; Left: *p* = 0.003) as well as reductions in peak knee valgus moments (Right: *p* = 0.033; Left: *p* = 0.013). There were no changes in peak knee extension moments (Right: *p* = 0.216; Left: *p* = 0.261). Increasing levels of breast support were associated with greater trunk flexion angles at initial contact (*p* = 0.024) and greater peak trunk flexion angles (*p* = 0.002).

**Conclusions:**

Lower levels of breast support are associated with knee joint and trunk biomechanical profiles suggested to increase ACL injury risk.

## Introduction

Landing tasks in multidirectional sports are associated with a variety of lower extremity injuries for both males and females. However, female athletes have a greater prevalence of traumatic knee injury than males (Arendt and Dick, 1995; NFHS, 2016). Specifically, female athletes are up to eight times more likely to experience an anterior cruciate ligament (ACL) injury than their male counterparts in the same sport (Arendt and Dick, 1995; NFHS, 2016).

The exaggerated rate of ACL injury in female athletes has been attributed in part to distinct differences in lower extremity biomechanical patterns in females compared to males. Pappas et al. (2007) revealed that females exhibit greater peak knee valgus angles and peak vertical ground reaction forces (GRFs) than male athletes (Pappas et al., 2007) when landing from a height of 40 cm. As the mechanical demand increased from 40 to 60 cm, female athletes also exhibited greater peak ankle dorsiflexion, and peak foot pronation than male athletes (Kernozek et al., 2005). Further, during unanticipated side-step cutting, females exhibited greater knee abduction angles at initial contact (IC) and greater peak ankle eversion angles during stance phase than males. Greater ankle eversion angle has been suggested to contribute to greater tibial internal rotation while greater knee valgus prior to cutting may place greater load on the structures of the knee including the ACL, therefore, increasing the risk of injury (Ford et al., 2005). These sex-related differences in lower extremity biomechanics during both landing and cutting may explain the greater rate and incidence of ACL injuries in female compared to male athletes.

An understudied factor known to alter lower extremity biomechanics and ACL injury risk is trunk biomechanics. During a sixty-centimeter vertical double-leg drop-landing task, individuals that landed with greater trunk flexion angles also exhibited greater hip and knee flexion angles (Blackburn and Padua, 2008, 2009). In addition, individuals landing with greater trunk, hip, and knee flexion angles also experienced a decrease in quadricep activity and increase in hamstring muscle force (Blackburn and Padua, 2009; Kulas et al., 2010). This increase in hamstring muscle force is suggested to counteract knee anterior shear forces when landing with greater trunk flexion, as opposed to landing with greater trunk extension (Kulas et al., 2010). Even when the mechanical demand of the task is decreased to a single-leg squat task, individuals squatting with a moderate amount of trunk lean flexion, as opposed to minimal amounts of trunk flexion, still experienced higher hamstring muscle forces and lower peak and mean ACL forces and strains (Kulas et al., 2012). Further, a study investigating the effect of fatigue on landing biomechanics demonstrated that sagittal and frontal plane trunk and lower extremity alignment are altered by fatigue potentially increasing risk of ACL injury during a landing task (Liederbach et al., 2014). While trunk biomechanics may play a role in increased ACL stress and increased ACL injury risk, these studies do not compare trunk biomechanical differences between females and males.

A sex-specific trait that has been shown to alter trunk biomechanics during sport-related movements is the female breast. Breast development occurs with physical maturation (Biro et al., 2013), has been associated with altered lower extremity biomechanics (Hewett et al., 2004; Sigward et al., 2012) and mirrors the sex-based divergence in ACL injury rates (Sanders et al., 2016). Female breasts are a passive tissue that are only supported by connective tissue (Gaskin et al., 2020). Because of this, breasts have limited intrinsic support and often require the use of extrinsic support, typically in the form of sports bras during highly dynamic activities. Without the use of sports bras and sufficient breast support, females can experience increased levels of embarrassment, decreased willingness to exercise, and increased levels of breast discomfort or pain (Risius et al., 2017). By wearing sports bras and sufficient support, females can control for vertical, anteroposterior, and mediolateral breast displacement (Scurr et al., 2009, 2011). Additionally, breast support has been found to create significant changes in running biomechanics including peak pelvis rotation, pelvis range of motion, vertical trunk oscillation, peak trunk rotation, and trunk range of motion as well as peak torso range of motion across all planes of motion (Milligan et al., 2015; Risius et al., 2017). However, a majority of breast support in sports movement research has limited focus to upper extremity and trunk biomechanics specifically during running. Further, this research has primarily investigated large breasted females with a breast size of a D-cup.

While previous literature has determined that both lower extremity and trunk biomechanics can increase the risk of ACL injuries, no previous research has investigated the effect of breast support on lower extremity and trunk biomechanics associated with ACL injury during landing tasks. Therefore, the purpose of this study is to determine the effect of sports bra support on trunk and knee joint biomechanics in female collegiate athletes during a double-leg landing task. It was hypothesized that increasing levels of breast support would be associated with knee and trunk biomechanics less indicative of ACL injury risk.

## Materials and Methods

### Participants

An *a prior* power analysis (G^*^Power 3.1.5) was conducted based on findings from preliminary data. Using an effect size of 0.40, an alpha level of 0.05 and power (1–β) of 0.80, it was determined that a total sample size of 12 will provide sufficient statistical power for the study (Portney and Watkins, 2009). A total of 14 female athletes were recruited for this study. However, *two* participants did not complete all experimental conditions, and were consequently not included in the data analysis. Inclusion criteria included (1) 18–25 years of age, (2) current or former (<2 years) female collegiate athlete, (3) self-reported bra size of B-DD cup, (4) no history of prior breast surgeries (reduction or implants), (5) free from a recent history of musculoskeletal injuries (within the past six months), and (6) free from any history of ACL injuries. The experimental protocol (PRO-FY2020-24) was approved by the University of Memphis Institutional Review Board and all participants provided written informed consent prior to data collection.

### Experimental Equipment

**Figure 1:**
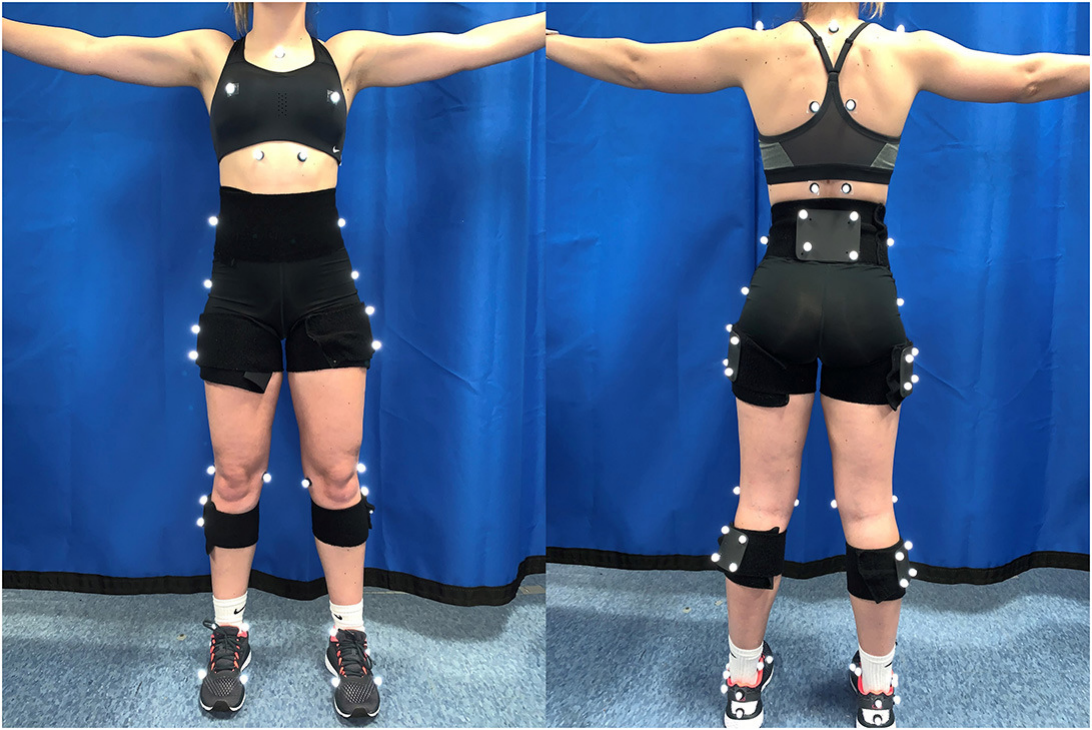
Image of retroreflective marker locations used to define and trunk the skeleton including the trunk, pelvis, right and left thigh, shank and foot. Anatomical markers including the left and right iliac crests, greater trochanters, medial and lateral femoral epicondyles and medial and lateral malleoli as well as the first and fifth metatarsals were removed prior to dynamic testing.

Participants were asked to wear spandex shorts and their preferred athletic shoes for testing. Ground reaction forces (GRFs) and three-dimensional kinematics were recorded simultaneously using a 10-camera motion capture system (250 Hz, Qualisys AB, Goteburg, Sweden) and two force platforms (1,500 Hz, AMTI Inc., Watertown, MA, USA) embedded in the laboratory floor. The skeleton was modeled using 14 mm retro-reflective markers and included trunk and pelvis, as well as left and right thigh, shank, and foot segments. Retro-reflective markers were placed bilaterally on the participant's lower extremity and trunk in order to track individual segment motion during the double-leg landing task. The pelvis, thigh, and shank were tracked using rigid clusters of four retroreflective markers. The rearfoot was tracked using three individual retroreflective markers placed over the superior, inferior and lateral calcaneus. The trunk was defined using individual markers placed over the left and right acromion processes and the right and left iliac crests (Figure 1). The trunk segment was tracked using individual markers placed on the skin over the superior sternum, the spinous process of the first thoracic vertebra (T1), the left and right transverse processes of the sixth thoracic vertebrae (T6), the left and right transverse processes of the 12th thoracic vertebra (T12) and the anterior portion of the 10th osteochondral junction. Breast motion was tracked using individual markers placed over the superior sternum and left and right nipples. Anatomical markers were placed over the left and right iliac crest, and trochanters. Anatomical markers were also be placed over the medial and lateral femoral epicondyles, medial and lateral malleoli, and the first and fifth metatarsal heads. After a standing calibration, anatomical markers were removed leaving only the tracking markers on the breast, trunk, pelvis, thigh, shank, and rearfoot.

### Experimental Protocol

Participants visited the Exercise Neuromechanics Research Laboratory at the University of Memphis once for examination and testing. Participants were screened for inclusion criteria, completed a written Physical Activity Readiness Questionnaire (PAR-Q), and provided written informed consent. Each testing session occurred in the following order: (1) measurement of anthropometric variables including age, height (cm), weight (kg), breast size (cm), and rib cage size (cm), (2) warm-up exercises, (3) placement of measurement sensors, and (4) completion of the dynamic testing protocol. The dynamic testing protocol consisted of a double-leg step-off landing task in each of three breast support conditions including low support (LOW), high support (HIGH), and no support (CON).

**Figure 2:**
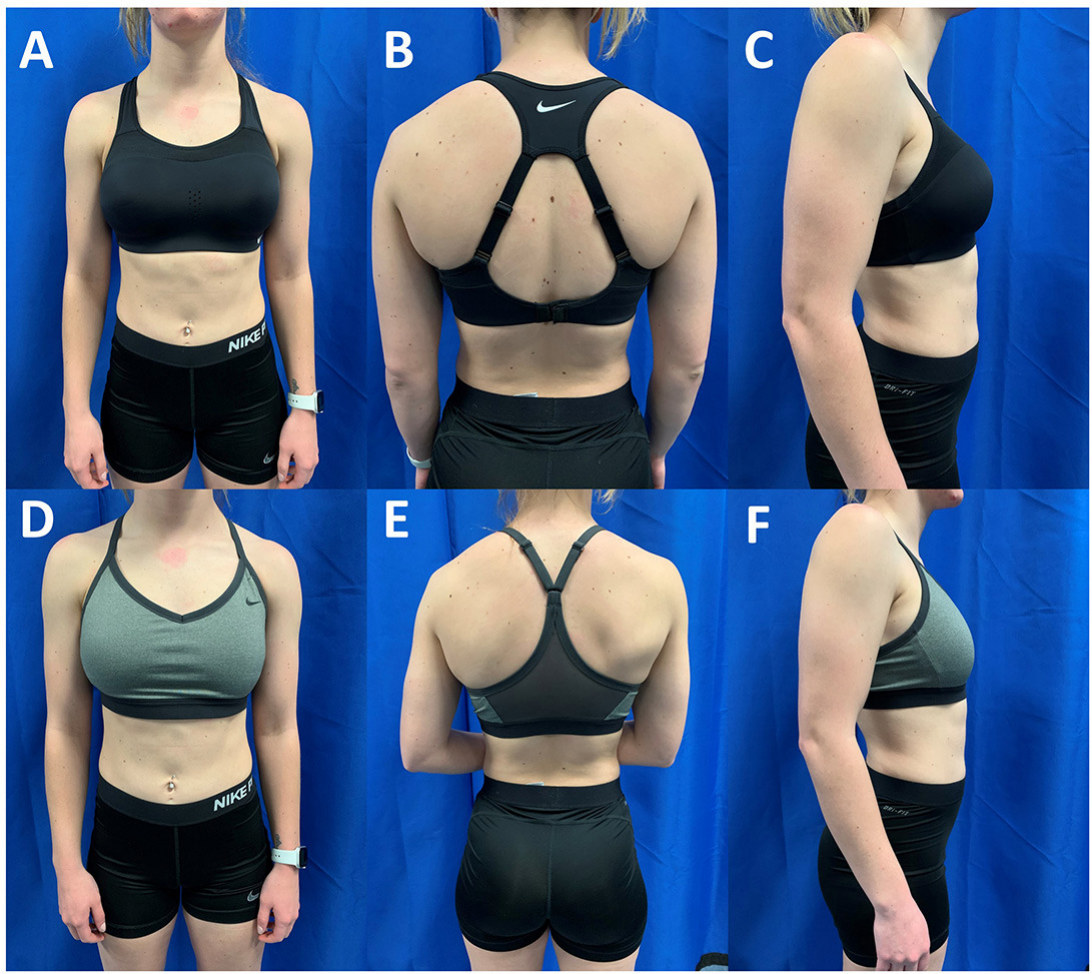
Anterior, posterior and lateral views of a participant with D-Cup sized breasts in the high support Nike Alpha **(A–C)** and low support Nike Indy **(D–F)**. The athlete was classified as a D-Cup based on the difference between her bust and underbust circumferences (Bust: 84 cm; Underbust: 73.5 cm; Difference: 10.5 cm). The high support sports bra is designed to lift and compress the breast tissue while the low support sports bra is not designed with these features.

The LOW conditions required the participant to wear a sports bra that is described by the manufacturer as having “light” support for low-impact workouts. The low support sports bras offered the breasts limited support. The low support sports bra was the Nike Indy (Nike Inc., Beaverton, OR, USA). The fabric of the sports bra includes a body and lining made of 88% recycled polyester and 12% spandex, center back mesh and bottom hem made of 81 percent nylon and 19 percent spandex, elastic made 84 to 85% nylon and 15 to 16% spandex, interlining made of 80% polyester and 20% spandex, pad top fabric and pad back fabric made of 100% polyester, and pad made of 100 percent polyurethane. The HIGH condition required the participant to wear a sports bra that is described by the manufacturer has having their “highest” level of support with a compressive feel for minimal bounce. The high support sports bra was the Nike Alpha (Nike Inc., Beaverton, OR, USA). The fabric of the sports bra includes a body and back lining insets made of 79% nylon and 21% spandex, mesh and mesh lining made of 81% nylon and 19% spandex, pad made of 100-polyurethane, and pad back fabric made of 100 percent polyester. The CON condition required the participant to complete the protocol bare chested with no sports bra and no breast support. CON condition was optional for participants. The purpose of the control condition is to compare data from previous studies to the current study. Sizes of the low and high support sports bras were determined based on fitting described by the manufacturer. Figure 2 depicts the breast support provided by each sports bra in a representative participant. The protocol was repeated in each randomized support condition (LOW and HIGH) while the CON condition was completed last.

The protocol consisted of a double-leg landing task which required the participant to step-off of a 40-cm box and land bilaterally with one foot on each force platform. A box height of 40 cm was selected as this height is commonly within the maximum vertical jump height of most female athletes. A successful trial was characterized by the participant landing from the box with simultaneous left and right ground contacts with one foot on each of the two force platforms. For foot contact, simultaneous ground contact was defined as having both feet strike the force platforms within a two frame (8 ms) window. Participants completed a total of five successful trials. The participants were allowed to familiarize themselves with the landing task for a period of several minutes until they reported their comfort. Participants performed the familiarization protocol prior to each support condition.

### Data Analysis

Landing data were analyzed from IC to an instant 100 ms after contact (INI). The energy absorbed during this period has been associated with injury biomechanics (Norcross et al., 2010) and includes the period in which the ACL is most likely to experience significant injury (Krosshaug et al., 2007; Koga et al., 2010; Bates et al., 2020). IC was determined as the instant at which vertical GRF exceeds a threshold of 20 N and remained above this threshold for a period >0.010 s. A 20 N threshold was selected as it represented a value more than 3 standard deviations above the mean baseline GRF value while the 0.010 s duration represented four frames of data and 10% of the window of analysis, thereby removing potential artifacts being identified as events of interest. Visual 3D (C-Motion Inc., Bethesda, MD, USA) was used to create a six degree-of-freedom kinematic model as well as filter kinematic and GRF data. The six degree-of-freedom model allows each modeled segment to move independently in three translational and three rotational directions. Retroreflective marker trajectories and GRF data were filtered using a fourth-order, zero-lag Butterworth lowpass filter with cutoff frequencies of 10 and 40 Hz, respectively (Smith et al., 2020). Sagittal and frontal plane knee joint angles and moments as well as sagittal plane trunk segment angles were calculated using Visual 3D (C-Motion Inc., Germantown, MD). Vertical breast displacement was calculated as the difference in position of the nipple markers relative to the position of the sternum marker within the plane of the trunk from IC to INI. The adjustment of the local axis system allows for the calculation of vertical breast displacement relative to the trunk. Custom software (MATLAB 2021a, MathWorks, Natick, MA) was used to calculate vertical breast displacement, sagittal and frontal plane knee joint angles at IC and at INI as well as peak sagittal and frontal plane knee joint moments between IC and INI.

### Statistical Analysis

A 1 x 3 (task by breast support level) repeated measures analysis of covariance (ANCOVA) was used to determine the effect of breast support level on knee joint and trunk biomechanics while controlling for the effect of breast size. Breast size was quantified as the difference between bust and underbust circumferences (in cm). An ANCOVA was selected to control for the potential effects of breast size on knee joint and trunk biomechanics.

In the presence of a significant main effect of support level, *post-hoc* pairwise comparisons were performed using paired samples *t*-tests to determine source of the significant interaction. A Holm-Bonferroni Correction was performed to adjust the level of significance for multiple comparisons (Holm, 1979). To conduct this correction, the *p*-values for *post-hoc* pairwise comparisons were placed in ascending order (from smallest to largest) and compared to the adjusted level of significance. As three pairwise comparisons were performed, significance for the first *post-hoc* comparison was set at *p* < 0.017 (*p* < 0.05/3) while significance for the second *post-hoc* comparison was set at *p* < 0.025 (*p* < 0.05/2) and significance for the third *post-hoc* comparison was set at *p* < 0.05 (*p* < 0.05/1). The sequential adjustment of the *p*-value is designed to reduce the risk of Type I error associated with multiple comparisons while also maintaining sufficient statistical power. Cohen's d estimates of effect sizes were also reported to further evaluate the effect of breast support on trunk and knee joint biomechanics (Cohen, 1988). Cohen's d values were interpreted as follows: small, d <0.2; moderate, 0.2 < d <0.8; large, d > 0.8. Significance for omnibus testing was set at *p* < 0.05 while *post-hoc* alpha levels were adjusted as previously described. All statistical comparisons were conducted using SPSS (IBM, Armonk, New York).

## Results

### Participants

Table 1 presents a summary of participant anthropometrics. Participants had an average age of 20.9 (± 1.7) years, average height of 170.1 (± 6.4) cm, average weight of 63.8 (± 6.9) kg, average bust circumference of 83.9 (± 2.4) cm, and average rib cage circumference of 74.3 (± 3.1) cm. No comparisons were made between individuals of different breast sizes.

**Table 1:** Participant anthropometric values including age, height, weight, bust and underbust circumferences, breast size and sport participation.

**Subject**	**Age**	**Height (cm)**	**Mass (kg)**	**Bust (cm)**	**Underbust (cm)**	**Breast size (cm)**	**Breast size (Cup)**	**Sport**	**Note**
S1	24	162	56.6	83	71.5	11.5	D	Track and Field	
S2	21	165	60.8	84	73.5	10.5	D	Soccer	
S3	21	164	53.5	82.5	73	9.5	C	Soccer	
S4	19	167	65	86.5	70	16.5	D	Soccer	Removed
S5	19	172.2	60.6	80	71.5	8.5	C	Soccer	
S6	23	172	65.8	85.5	78	7.5	B	Track and Field	
S7	22	165.1	56.3	79.5	72.5	7	B	Soccer	
S8	23	172	59.87	82.5	76	6.5	B	Volleyball	Removed
S9	21	163.1	60.6	85	72.5	12.5	D	Soccer	
S10	20	181.2	73.3	82.5	75.5	7	B	Volleyball	
S11	20	178.4	74.7	86	80.5	5.5	B	Volleyball	
S12	18	167.7	73.6	88	78.5	9.5	C	Volleyball	
S13	20	181	70.5	83.5	75.5	8	C	Volleyball	
S14	21	170.3	61.5	85.5	71.5	14	D	Softball	
Mean	20.8	170.2	64.0	83.8	74.5	9.3			
SD	1.6	6.9	7.4	2.5	3.1	2.5			

### Breast Displacement

**Figure 3:**
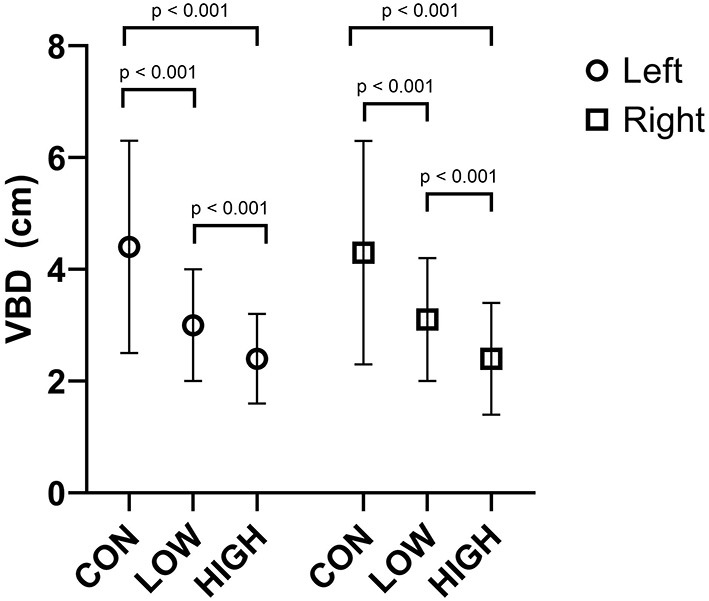
Average vertical breast displacement (VBD) of the left and right breast in the CON, LOW and HIGH support conditions during the double-leg landing task. Displacements are presented in cm.

Increasing levels of breast support were associated with reductions in vertical breast displacement (Figure 3) during the double-leg landing task for the left (*F* = 3.0, *p* < 0.001) and right breasts (*F* = 3.4, *p* < 0.001). Breast displacement was greater in the CON compared to LOW (Left: *p* < 0.001, *d* = 0.92; Right: *p* < 0.001, *d* = 0.74) and HIGH (Left: *p* < 0.001, *d* = 1.37; Right: *p* < 0.001, *d* = 1.20) breast support conditions while breast displacement was also greater in the LOW compared to HIGH support conditions (Left: *p* < 0.001, *d* = 0.66; Right: *p* < 0.001; *d* = 0.067).

### Knee Joint Angles

At IC, level of sports bra support was not associated with changes in knee flexion angles for either left (*F* = 1.25; *p* = 0.166) or right (*F* = 1.42; *p* = 0.146) legs. Moreover, no effect of sports bra support was observed for knee joint valgus angles for either left (*F* = 0.60; *p* = 0.284) or right (*F* = 0.65; *p* = 0.284) legs.

At INI, level of sports bra support was associated with altered knee joint flexion angles for both left (*F* = 3.40; *p* = 0.029) and right (*F* = 6.94; *p* = 0.008) legs (Table 2). For the left leg, no differences were observed in knee flexion angles at INI between the CON and LOW conditions (*p* = 0.370, *d* = 0.72) or the LOW and HIGH conditions (*p* = 0.167, *d* = 0.27) while CON condition was associated with greater knee flexion angles than the HIGH condition (*p* = 0.039, *d* = 0.58). For the right leg, knee flexion angles at INI were greater in the CON compared to the LOW (*p* = 0.009, *d* = 0.50) and HIGH conditions (*p* = 0.019, *d* = 0.52). However, no differences were observed between the LOW and HIGH conditions (*p* = 0.493, *d* = 0.01).

**Table 2:** Knee joint kinematics during the double-limb landing task.

**Limb**	**Condition**	**Flexion Angle at IC (**°**)**	**Valgus Angle at IC (**°**)**	**Flexion Angle at INI (**°**)**	**Valgus Angle at INI (**°**)**
Left	Control	19.2 ± 4.4	−0.4 ± 3.9	68.8 ± 4.3	−5.1 ± 6.9
	Low	20.4 ± 6.9	0.5 ± 2.9	67.6 ± 7.0	−2.0 ± 6.1^a^
	High	17.9 ± 4.7	0.7 ± 2.8	66.2 ± 4.7^a^	−0.2 ± 6.0^a^
	*p*-value	0.166	0.284	**0.029**	**0.003**
Right	Control	19.4 ± 4.8	−0.7 ± 2.6	69.0 ± 4.9	−6.5 ± 5.3
	Low	18.3 ± 5.9	0.6 ± 3.2	66.3 ± 5.8^a^	−2.1 ± 6.7^a^
	High	18.5 ± 5.4	0.9 ± 2.0	66.3 ± 5.5^a^	−0.4 ± 4.2^a^
	p-value	0.146	0.284	**0.008**	**0.011**

a *Denotes significant difference compared to CON support condition*.

*Presented as mean ± SD. Bold values represent statistical significance (p <0.05)*.

Knee valgus angles at INI (Table 2) were altered by increasing levels of sports bra support for both left (*F* = 11.01; *p* = 0.003) and right (*F* = 11.0; *p* = 0.011) legs. The CON condition was associated with greater knee valgus angles than either the LOW (Left: *p* = 0.002, *d* = 0.48; Right: *p* = 0.003, *d* = 0.73) or HIGH conditions (Left: *p* = 0.001, *d* = 0.76; Right: *p* = 0.003, *d* = 1.28). No differences in knee valgus angles were observed between the LOW and HIGH conditions (Left: *p* = 0.355, *d* = 0.30; Right: *p* = 0.362, *d* = 0.30).

### Knee Joint Moments

**Figure 4:**
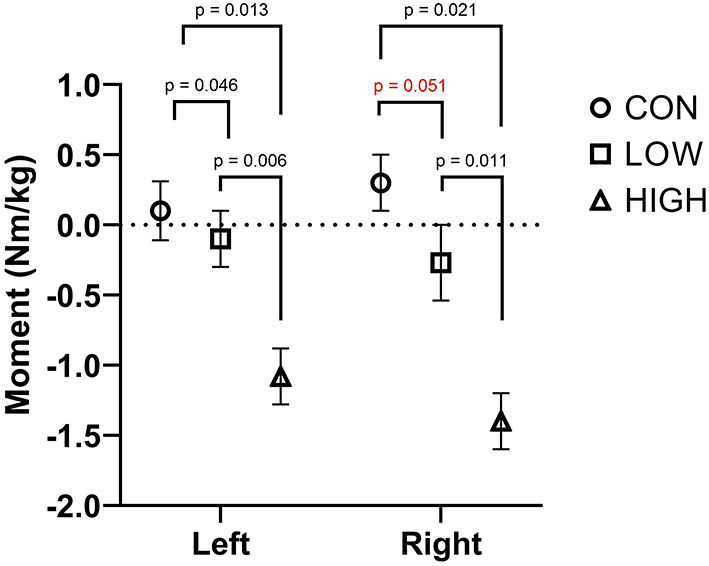
Peak knee extension moments of the left and right leg in the CON, LOW and HIGH support conditions during the double-leg landing task. Joint moments are presented in Nm/kg.

Figure 4 presents peak knee joint extension moments during the double leg landing task. Level of sports bra supports had no effect on peak knee joint moments for left (*F* = 0.96; *p* = 0.216) or right (*F* = 4.22; *p* = 0.261) legs.

**Figure 5:**
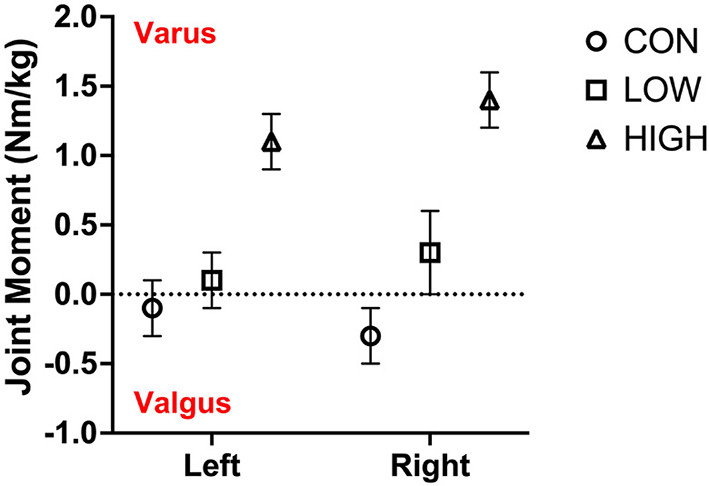
Peak knee valgus moments of the left and right leg in the CON, LOW and HIGH support conditions during the double-leg landing task. Joint moments are presented in Nm/kg.

Peak valgus moments were reduced with greater levels of breast support during the double-leg landing task (Figure 5). For the left leg, peak knee valgus moments were reduced with increasing breast support (*F* = 3.91; *p* = 0.033). *Post-hoc* comparisons revealed greater knee valgus moments in the CON compared to LOW (*p* = 0.046, *d* = 0.98) and HIGH (*p* = 0.013, *d* = 5.75) while the LOW was associated with greater knee valgus moments than the HIGH (*p* = 0.006, *d* = 4.90). For the right leg, increasing levels of breast support were associated with smaller peak knee valgus moments (*F* = 4.00; *p* = 0.038). Pairwise comparisons revealed no differences between the CON and LOW conditions (*p* = 0.051, *d* = 2.40) while the CON was associated with greater peak knee valgus moments than the HIGH support condition (*p* = 0.021, *d* = 8.5). Further, the LOW condition was associated with greater peak knee valgus moments than the HIGH condition (*p* = 0.011, *d* = 4.76).

### Trunk Angles

At IC, increasing levels of breast support were associated with greater trunk flexion (Table 3; *F* = 4.59; *p* = 0.024). *Post-hoc* analyses revealed no differences in trunk flexion angles between the CON and LOW support conditions (*p* = 0.142, *d* = 0.24) while trunk flexion angles were greater in the HIGH compared to CON (*p* = 0.006, *d* = 0.53) and LOW support conditions (*p* = 0.020, *d* = 0.29). Similarly, increasing levels of breast support were associated with greater trunk flexion at INI (*F* = 15.3; *p* = 0.001). Pairwise comparisons demonstrated that trunk flexion angles were greater in the LOW (*p* = 0.001, *d* = 0.58) and HIGH conditions (*p* = 0.001, *d* = 0.99) compared to CON condition while trunk flexion angles were greater in the HIGH compared to LOW support conditions (*p* = 0.003, *d* = 0.38).

**Table 3:** Trunk angles at IC and at INI during the double-limb landing task as well as the statistical results of the omnibus ANCOVA.

**Event**	**CON**	**LOW**	**HIGH**	***p*-value**
IC	−0.5 ± 2.5	0.1 ± 2.5	0.8 ± 2.4^a, b^	**0.024**
INI	−1.4 ± 1.8	−0.2 ± 2.3^a^	0.7 ± 2.4^a^^,^ ^b^	**0.002**

a *Denotes significant difference compared to CON support condition*.

b *Denotes significant difference compared to the LOW support condition*.

*Presented as mean ± SD*.

## Discussion

The purpose of the current study was to determine the effects of breast support level on knee joint and trunk biomechanics in female collegiate athletes during a double-leg landing task. The major findings of this study were that increasing levels of breast support were associated with smaller peak knee flexion angles, smaller peak knee valgus angles and smaller peak knee valgus moments. Further, greater breast support was also associated with greater trunk flexion at IC and greater peak trunk flexion during the first 100 ms following ground contact.

Knee joint flexion is a major contributor to load attenuation during a landing task (Zhang et al., 2008). The current findings demonstrated that greater levels of breast support were associated with reduced knee flexion and knee flexion excursions within the first 100 ms following IC. When considering the lower extremity as a linear spring, knee flexion is a dampening movement (Farley and Morgenroth, 1999; Powell et al., 2014, 2016, 2017). It is postulated that greater knee flexion observed in the low support conditions (CON and LOW) represents a neuromuscular strategy associated with less leg stiffness (greater compliance) which would decelerate the pelvis and trunk along with the passive breast tissue over a longer period of time, decreasing the vertical accelerations of the breast tissue to reduce breast pain. Conversely, in the high support condition, the breast tissue was constrained by the sports bra reducing breast accelerations during the landing task which allowed the participants to land with a preferred landing pattern with greater stiffness. Increased leg stiffness has been suggested to be indicative of better athletic performance (Butler et al., 2003).

Though knee flexion excursions were reduced with increasing breast support, no differences were observed in peak knee extension moments between the breast support conditions. When considering the lower extremity as a torsional spring, the combination of similar knee extension moments and reduced knee flexion excursions would result in greater knee joint stiffness and greater joint loading during the landing task (Butler et al., 2003; Powell et al., 2017). Previous research has shown that greater joint stiffness values are associated with greater vertical loading rates (Butler et al., 2003; Williams et al., 2004; Powell et al., 2017) and greater peak vertical ground reaction forces (Butler et al., 2003; Williams et al., 2004; Powell et al., 2017; Arnwine and Powell, 2020), each of which is associated with an increased risk of musculoskeletal injury (Whiting and Zernicke, 1998). Due to the short duration of the analysis period following IC (100 ms), the biomechanics of the landing task were the result of a predicted mechanical requirement of the landing task and were not the result of a feedback dominant motor pattern. Evidence has demonstrated that long latency reflex control (involving sensory processing by supraspinal structures) of lower leg muscle activation presents with latencies >100 ms (Tsuda et al., 2001, 2003). Therefore, we propose that the greater knee flexion excursions associated with the low breast support conditions (CON and LOW) were the result of a predictive motor control pattern selected to increase lower leg compliance and reduce accelerations of the passive breast tissue during the landing task.

A secondary outcome of greater knee flexion and leg compliance in the lower breast support conditions (CON and LOW) during the landing task is an expansion of the available knee joint range of motion in the frontal and transverse planes (Nordin and Frankel, 2012). The current data demonstrated that in the low breast support conditions (CON and LOW), peak knee valgus angles were greater than in the HIGH breast support condition. Greater knee valgus during a landing task has been associated with reduced neuromuscular control and a greater risk of ACL injury (Hewett et al., 2005; Kernozek et al., 2005; Pappas et al., 2007). Though the differences in knee valgus angles at INI between breast support conditions were small (~3°-4°), research has suggested that deviations in frontal plane knee joint angle as small as 2° can result in meaningful reductions in the external load required to rupture the ACL (Chaudhari and Andriacchi, 2006). The mechanical effect of greater knee valgus angles is supported by the current findings which demonstrated reduced knee valgus moments in the greater breast support conditions.

Trunk motion has been suggested to modify knee joint biomechanics during load attenuation tasks including single leg squatting and landing tasks (Blackburn and Padua, 2008, 2009; Kulas et al., 2010, 2012). Using a modeling approach, Kulas et al. (2012) revealed that a moderate forward trunk lean was associated with lower peak ACL forces and strains compared to a minimal forward trunk lean during a single-leg squat. Similarly, during a double-leg landing, Kulas et al. (2010) demonstrated that individuals that land with moderate trunk flexion exhibit less knee anterior shear forces and greater hamstrings muscle forces compared to individuals that land with an extended trunk position. Functionally, the hamstrings muscle group acts to protect the ACL by limiting anterior translation of the tibia relative to the femur. Moreover, an intrinsic ACL-hamstrings reflex pathway exists to provide active, muscular support to an ACL that is experiencing strain (Tsuda et al., 2001). The findings of the current study demonstrate that greater breast support was associated with increased trunk flexion angles at IC as well as peak trunk flexion angles. Therefore, these data suggest that the high breast support condition was associated with trunk biomechanics that are indicative of a lower risk of ACL injury compared to low breast support conditions (CON or LOW).

While the current study presents novel findings pertaining to the influence of breast support on knee joint and trunk biomechanics, the authors acknowledge several limitations of the current study. One limitation of the current study is the assumption that the participants are wearing the correct sports bra size and therefore the sports bra support level based on their self-known bra size. However, research has suggested that up to 80% of females are wearing the incorrect bra size (McGhee and Steele, 2010; Hupprich et al., 2020). While the participants were measured for the “correct” bra size using bust and rib cage circumferences, this technique has been criticized for its inaccuracies in measuring for bra size (McGhee and Steele, 2011). However, this specific technique to measure for bra size is commonly used and feasible for the entire female population, which is why it was used. A second limitation of the current study is the small sample size with the power analysis suggesting only 12 participants. The small sample size of only 12 participants may injure any generalizations made to the population. However, even with this small sample size, the study was sufficiently powered to find significant differences between breast support conditions. Furthermore, while 12 participants were required for the power analysis, not all participants completed the CON condition which limited comparisons. Therefore, an additional two participants were recruited to reach the required number of 12, and a total of 14 participants were collected. Another limitation of the current findings pertains to the measurement accuracy of motion capture systems in frontal plane kinematics. Specifically, it is known that skin artifact can negatively affect accuracy of marker-based motion capture systems (Chiari et al., 2005; Leardini et al., 2005). Further, these errors disproportionately effect frontal and transverse plane kinematic calculations. As such, the current findings should be view in light of these limitations in motion capture.

## Conclusions

Greater breast support was associated with a multi-joint biomechanical adaptation characterized by reduced knee flexion, reduced knee valgus and greater trunk flexion angles. These movement profiles are associated with lower risks of traumatic knee injury suggesting that breast support is an important consideration for optimal sport performance and injury prevention. Future research should expand the current analysis to investigate altered contributions of the ankle and hip joint as well. Moreover, lower extremity stiffness and its interaction with trunk biomechanics should also be investigated.

## Data Availability Statement

The raw data supporting the conclusions of this article will be made available by the authors, without undue reservation.

## Ethics Statement

The studies involving human participants were reviewed and approved by University of Memphis Institutional Review Board (PRO-FY2020-24). The patients/participants provided their written informed consent to participate in this study.

## Author Contributions

HF, AN, JS, and JH collected data and prepared the initial draft of the manuscript. HF, AN, JS, JH, and DP performed data analysis. All authors were involved in study conception and design. All authors revised, edited and approved the final manuscript.

## Funding

This project received financial support from the American Athletic Conference Research Consortium.

## Conflict of Interest

The authors declare that the research was conducted in the absence of any commercial or financial relationships that could be construed as a potential conflict of interest.

## Publisher's Note

All claims expressed in this article are solely those of the authors and do not necessarily represent those of their affiliated organizations, or those of the publisher, the editors and the reviewers. Any product that may be evaluated in this article, or claim that may be made by its manufacturer, is not guaranteed or endorsed by the publisher.

## References

[B1] Arendt E. Dick R. (1995). Knee injury patterns among men and women in collegiate basketball and soccer. NCAA data and review of literature. Am. J. Sports Med. 23, 694–701. 10.1177/036354659502300611 8600737

[B2] Arnwine R. A. Powell D. W. (2020). Sex differences in ground reaction force profiles of ballet dancers during single- and double-leg landing tasks. J. Dance Med. Sci. 24, 113–117. 10.12678/1089-313X.24.3.113 32867913

[B3] Bates N. A. Schilaty N. D. Ueno R. Hewett T. E. (2020). Timing of strain response of the ACL and MCL relative to impulse delivery during simulated landings leading up to ACL failure. J. Appl. Biomech. 10.1123/jab.2019-0308. [Epub ahead of print]. 32320947 PMC7764947

[B4] Biro F. M. Greenspan L. C. Galvez M. P. Pinney S. M. Teitelbaum S. Windham G. C.. (2013). Onset of breast development in a longitudinal cohort. Pediatrics 132, 1019–1027. 10.1542/peds.2012-3773 24190685 PMC3838525

[B5] Blackburn J. T. Padua D. A. (2008). Influence of trunk flexion on hip and knee joint kinematics during a controlled drop landing. Clin. Biomech. (Bristol, Avon) 23, 313–319. 10.1016/j.clinbiomech.2007.10.003 18037546

[B6] Blackburn J. T. Padua D. A. (2009). Sagittal-plane trunk position, landing forces, and quadriceps electromyographic activity. J. Athl. Train. 44, 174–179. 10.4085/1062-6050-44.2.174 19295962 PMC2657019

[B7] Butler R. J. Crowell H. P. III. Davis I. M. (2003). Lower extremity stiffness: implications for performance and injury. Clin. Biomech. (Bristol, Avon) 18, 511–517. 10.1016/S0268-0033(03)00071-8 12828900

[B8] Chaudhari A. M. Andriacchi T. P. (2006). The mechanical consequences of dynamic frontal plane limb alignment for non-contact ACL injury. J. Biomech. 39, 330–338. 10.1016/j.jbiomech.2004.11.013 16321635

[B9] Chiari L. Della Croce U. Leardini A. Cappozzo A. (2005). Human movement analysis using stereophotogrammetry. Part 2: instrumental errors. Gait Posture 21, 197–211. 10.1016/j.gaitpost.2004.04.004 15639399

[B10] Cohen J.. (1988). Statistical Power Analysis for the Behavioral Sciences. New York, NY: Routledge Academic.

[B11] Farley C. T. Morgenroth D. C. (1999). Leg stiffness primarily depends on ankle stiffness during human hopping. J. Biomech. 32, 267–273. 10.1016/S0021-9290(98)00170-5 10093026

[B12] Ford K. R. Myer G. D. Toms H. E. Hewett T. E. (2005). Gender differences in the kinematics of unanticipated cutting in young athletes. Med. Sci. Sports Exerc. 37, 124–129. 10.1249/01.MSS.0000150087.95953.C3 15632678

[B13] Gaskin K. M. Peoples G. E. McGhee D. E. (2020). The attachments of the breast to the chest wall: a dissection study. Plast. Reconstr. Surg. 146, 11e−22e. 10.1097/PRS.0000000000006954 32590636

[B14] Hewett T. E. Myer G. D. Ford K. R. (2004). Decrease in neuromuscular control about the knee with maturation in female athletes. J. Bone Joint Surg. Am. 86, 1601–1608. 10.2106/00004623-200408000-00001 15292405

[B15] Hewett T. E. Myer G. D. Ford K. R. Heidt R. S. Jr. Colosimo A. J. McLean S. G.. (2005). Biomechanical measures of neuromuscular control and valgus loading of the knee predict anterior cruciate ligament injury risk in female athletes: a prospective study. Am. J. Sports Med. 33, 492–501. 10.1177/0363546504269591 15722287

[B16] Holm S.. (1979). A simple sequentially rejective multiple test procedure. Scand. J. Stat. 6, 65–70.

[B17] Hupprich M. R. Fong H. B. Nelson A. K. Hinton J. J. Puppa M. J. Powell D. W. (2020). “Bra sizing error in recreational and competitive athletes,” in Proceedings of the MidSouth Biomechanics Conference 3 (Memphis).

[B18] Kernozek T. W. Torry M. R. H V.A. N. H. Cowley H. Tanner S. (2005). Gender differences in frontal and sagittal plane biomechanics during drop landings. Med. Sci. Sports Exerc. 37, 1003–1012. discussion: 1013. 15947726

[B19] Koga H. Nakamae A. Shima Y. Iwasa J. Myklebust G. Engebretsen L.. (2010). Mechanisms for noncontact anterior cruciate ligament injuries: knee joint kinematics in 10 injury situations from female team handball and basketball. Am. J. Sports Med. 38, 2218–2225. 10.1177/0363546510373570 20595545

[B20] Krosshaug T. Nakamae A. Boden B. P. Engebretsen L. Smith G. Slauterbeck J. R.. (2007). Mechanisms of anterior cruciate ligament injury in basketball: video analysis of 39 cases. Am. J. Sports Med. 35, 359–367. 10.1177/0363546506293899 17092928

[B21] Kulas A. S. Hortobagyi T. Devita P. (2010). The interaction of trunk-load and trunk-position adaptations on knee anterior shear and hamstrings muscle forces during landing. J. Athl. Train. 45, 5–15. 10.4085/1062-6050-45.1.5 20064042 PMC2808754

[B22] Kulas A. S. Hortobagyi T. DeVita P. (2012). Trunk position modulates anterior cruciate ligament forces and strains during a single-leg squat. Clin. Biomech. (Bristol, Avon) 27, 16–21. 10.1016/j.clinbiomech.2011.07.009 21839557

[B23] Leardini A. Chiari L. Della Croce U. Cappozzo A. (2005). Human movement analysis using stereophotogrammetry. Part 3. Soft tissue artifact assessment and compensation. Gait Posture 21, 212–225. 10.1016/j.gaitpost.2004.05.002 15639400

[B24] Liederbach M. Kremenic I. J. Orishimo K. F. Pappas E. Hagins M. (2014). Comparison of landing biomechanics between male and female dancers and athletes, part 2: Influence of fatigue and implications for anterior cruciate ligament injury. Am. J. Sports Med. 42, 1089–1095. 10.1177/0363546514524525 24595401

[B25] McGhee D. E. Steele J. R. (2010). Optimising breast support in female patients through correct bra fit. A cross-sectional study. J. Sci. Med. Sport 13, 568–572. 10.1016/j.jsams.2010.03.003 20451452

[B26] McGhee D. E. Steele J. R. (2011). Breast volume and bra size. Int. J. Cloth. Sci. Technol. 23, 351–360. 10.1108/09556221111166284

[B27] Milligan A. Mills C. Corbett J. Scurr J. (2015). The influence of breast support on torso, pelvis and arm kinematics during a five kilometer treadmill run. Hum. Mov. Sci. 42, 246–260. 10.1016/j.humov.2015.05.008 26079773

[B28] NFHS (2016). National Federation of State High School Associations 2015 - 2016 High School Athletics Participation Survey. Available online at: http://www.nfhs.org/ParticipationStatistics/PDF/2015-16_Sports_Participation_Survey.pdf (accessed October 19, 2020).

[B29] Norcross M. F. Blackburn J. T. Goerger B. M. Padua D. A. (2010). The association between lower extremity energy absorption and biomechanical factors related to anterior cruciate ligament injury. Clin. Biomech. (Bristol, Avon) 25, 1031–1036. 10.1016/j.clinbiomech.2010.07.013 20797812

[B30] Nordin M. Frankel V. H. (2012). Basic Biomechanics of the Musculoskeletal System. Baltimore, MD: Lippincott Williams & Wilkins.

[B31] Pappas E. Hagins M. Sheikhzadeh A. Nordin M. Rose D. (2007). Biomechanical differences between unilateral and bilateral landings from a jump: gender differences. Clin. J. Sport Med. 17, 263–268. 10.1097/JSM.0b013e31811f415b 17620779

[B32] Portney L. G. Watkins M. P. (2009). Foundations of Clinical Research: Applications to Practice. Upper Saddle River, NJ: Pearson/Prentice Hall.

[B33] Powell D. W. Paquette M. R. Williams D. S. B. 3rd (2017). Contributions to leg stiffness in high- compared with low-arched athletes. Med. Sci. Sports Exerc. 49, 1662–1667. 10.1249/MSS.0000000000001279 28709154

[B34] Powell D. W. Queen R. M. Williams D. S. 3rd. (2016). Arch structure is associated with unique joint work, relative joint contributions and stiffness during landing. Hum. Mov. Sci. 49, 141–147. 10.1016/j.humov.2016.06.017 27391463

[B35] Powell D. W. Williams D. S. 3rd, Windsor, B. Butler R. J. Zhang S. (2014). Ankle work and dynamic joint stiffness in high- compared to low-arched athletes during a barefoot running task. Hum. Mov. Sci. 34, 147–156. 10.1016/j.humov.2014.01.007 24556475

[B36] Risius D. Milligan A. Berns J. Brown N. Scurr J. (2017). Understanding key performance indicators for breast support: an analysis of breast support effects on biomechanical, physiological and subjective measures during running. J. Sports Sci. 35, 842–851. 10.1080/02640414.2016.1194523 27291899

[B37] Sanders T. L. Maradit Kremers H. Bryan A. J. Larson D. R. Dahm D. L. Levy B. A.. (2016). Incidence of anterior cruciate ligament tears and reconstruction: a 21-year population-based study. Am. J. Sports Med. 44, 1502–1507. 10.1177/0363546516629944 26920430

[B38] Scurr J. White J. Hedger W. (2009). Breast displacement in three dimensions during the walking and running gait cycles. J. Appl. Biomech. 25, 322–329. 10.1123/jab.25.4.322 20095453

[B39] Scurr J. C. White J. L. Hedger W. (2011). Supported and unsupported breast displacement in three dimensions across treadmill activity levels. J. Sports Sci. 29, 55–61. 10.1080/02640414.2010.521944 21077006

[B40] Sigward S. M. Pollard C. D. Powers C. M. (2012). The influence of sex and maturation on landing biomechanics: implications for anterior cruciate ligament injury. Scand. J. Med. Sci. Sports 22, 502–509. 10.1111/j.1600-0838.2010.01254.x 21210853 PMC3117023

[B41] Smith R. E. Paquette M. R. Harry J. R. Powell D. W. Weiss L. W. (2020). Footwear and sex differences in performance and joint kinetics during maximal vertical jumping. J. Strength Cond. Res. 34, 1634–1642. 10.1519/JSC.0000000000002740 29979276

[B42] Tsuda E. Ishibashi Y. Okamura Y. Toh S. (2003). Restoration of anterior cruciate ligament-hamstring reflex arc after anterior cruciate ligament reconstruction. Knee Surg. Sports Traumatol. Arthrosc. 11, 63–67. 10.1007/s00167-002-0338-3 12664196

[B43] Tsuda E. Okamura Y. Otsuka H. Komatsu T. Tokuya S. (2001). Direct evidence of the anterior cruciate ligament-hamstring reflex arc in humans. Am. J. Sports Med. 29, 83–87. 10.1177/03635465010290011801 11206261

[B44] Whiting W. C. Zernicke R. F. (1998). Biomechanics of Musculoskeletal Injury. Champaign, IL: Human Kinetics.

[B45] Williams D. S. 3rd, Davis, I. M. Scholz J. P. Hamill J. Buchanan T. S. (2004). High-arched runners exhibit increased leg stiffness compared to low-arched runners. Gait Posture 19, 263–269. 10.1016/S0966-6362(03)00087-0 15125915

[B46] Zhang S. Derrick T. R. Evans W. Yu Y. J. (2008). Shock and impact reduction in moderate and strenuous landing activities. Sports Biomech. 7, 296–309. 10.1080/14763140701841936 18610780

